# Heart Failure With Preserved Ejection Fraction: A Practical, Stepwise Guide to Evidence-Based Therapy in 2026

**DOI:** 10.7759/cureus.115294

**Published:** 2026-08-27

**Authors:** M. Nour Chabalout, Mohammad Alazzeh, Dalia Othman, Jaswinder Pal Singh Virk

**Affiliations:** 1 Internal Medicine, Tower Health Medical Group, Phoenixville, USA; 2 Cardiology, Phoenixville Hospital, Phoenixville, USA

**Keywords:** glp1 receptor agonist, guideline-directed medical therapy (gdmt), heart failure with preserved ejection fraction (hfpef), innovative teaching strategies, sodium-glucose cotransporter-2 (sglt2) inhibitors

## Abstract

Heart failure with preserved ejection fraction (HFpEF) represents a large and growing share of the overall heart failure population and remains clinically challenging to manage. HFpEF has historically lacked effective therapies and a clear treatment algorithm accessible to non-specialists. Recent years have brought new pharmacologic options and expanded regulatory approvals, along with combination-therapy modeling suggesting meaningful projected lifetime benefit, yet translation of this evidence into everyday practice remains slow, particularly outside cardiology. Existing reviews synthesizing this evidence are predominantly specialist-oriented, lengthy, and organized by drug class rather than by clinical decision point, leaving a gap for the residents, hospitalists, internists, and primary care physicians who manage most patients with HFpEF. This technical report presents a concise, phenotype-guided, four-step treatment framework, informed by contemporary trial evidence and situated alongside current guideline pathways, together with a brief diagnostic checkpoint, a summary evidence table, and a discussion of common pitfalls, to facilitate rapid clinical application. This framework is an author-derived synthesis rather than a prospectively validated or guideline-endorsed treatment sequence.

## Introduction

Heart failure with preserved ejection fraction (HFpEF) management has undergone a paradigm shift. Within the past three years, multiple landmark trials have established new therapeutic options, regulatory approvals have expanded, and combination therapy modeling has demonstrated substantial projected lifetime benefits [1-3]. Yet the translation of this evidence into everyday clinical practice remains slow, particularly outside of cardiology.

HFpEF affects over 3 million individuals in the United States and is now the most common form of heart failure, a burden reflected in general reviews of the condition [4]. Management is now framed by the 2026 American College of Cardiology (ACC) Expert Consensus Decision Pathway (ECDP), which updates the 2023 version and designates an SGLT2 inhibitor plus a nonsteroidal mineralocorticoid receptor antagonist (MRA) as foundational therapy for most patients, with finerenone favored over spironolactone given its more favorable hyperkalemia profile [5]. Guideline recommendation strength for SGLT2 inhibitors in HFpEF varies by source and year: the 2022 AHA/ACC/HFSA heart failure guideline assigns a Class IIa recommendation specifically for HFpEF, while more recent guidance incorporating EMPEROR-Preserved and DELIVER data has moved toward stronger recommendations in some jurisdictions [6-8]. Finerenone received FDA approval in 2025 for reducing cardiovascular death, heart failure hospitalization, and urgent heart failure visits in adults with heart failure and left ventricular ejection fraction (LVEF) of 40% or higher, based on the FINEARTS-HF trial [1,9]. Glucagon-like peptide-1 (GLP-1) receptor agonists and dual GIP/GLP-1 receptor agonists have also demonstrated meaningful symptomatic and functional benefit in obesity-related HFpEF, and the 2026 ACC ECDP now recommends incretin-based therapy for patients with a body mass index of 30 kg/m² or higher [5,10].

Several high-quality reviews of HFpEF have been published recently, including a comprehensive review in the Lancet [4]. This review is cited here to establish the existing landscape of HFpEF literature against which this technical report is positioned: it is an invaluable, in-depth resource, but it is also organized by pathophysiology or drug class rather than by clinical decision point, and is written primarily for a cardiology audience. This characterization directly motivates the concise, decision-point-oriented format adopted below.

Meanwhile, the clinicians who manage the majority of patients with HFpEF, namely, primary care physicians, hospitalists, and trainees, face a rapidly evolving evidence base without a concise, actionable guide. The 2026 ACC ECDP itself is comprehensive and detailed, and while it is now the most current and authoritative source, its length and diagnostic complexity leave room for a shorter companion resource oriented specifically around point-of-care treatment sequencing [5].

This report addresses that gap by presenting an updated, stepwise, phenotype-guided treatment framework designed for rapid clinical application. This framework is an evidence-informed synthesis by the authors and is explicitly compared with the 2026 ACC ECDP throughout; it is not itself a prospectively validated or guideline-endorsed treatment sequence, a distinction discussed further below.

## Technical report

Before pharmacologic sequencing is considered, the diagnosis of HFpEF should itself be established, since this is frequently the most difficult step for non-specialists. At a minimum, this requires symptoms or signs of heart failure, an LVEF of 40% or higher, and objective evidence of a structural or functional cardiac abnormality or elevated natriuretic peptides. The 2026 ACC ECDP recommends sequential use of the H2FPEF and HFA-PEFF scoring systems for probability assessment, followed by systematic exclusion of noncardiac mimics (pulmonary disease, kidney disease, high-output states, obesity, frailty) and cardiac mimics (valvular disease, constrictive pericarditis, infiltrative cardiomyopathies such as cardiac amyloidosis, hypertrophic cardiomyopathy, and ischemic heart disease) before HFpEF is confirmed as a diagnosis of exclusion [5]. Only after this diagnostic checkpoint should the treatment framework below be applied.

**Figure 1:**
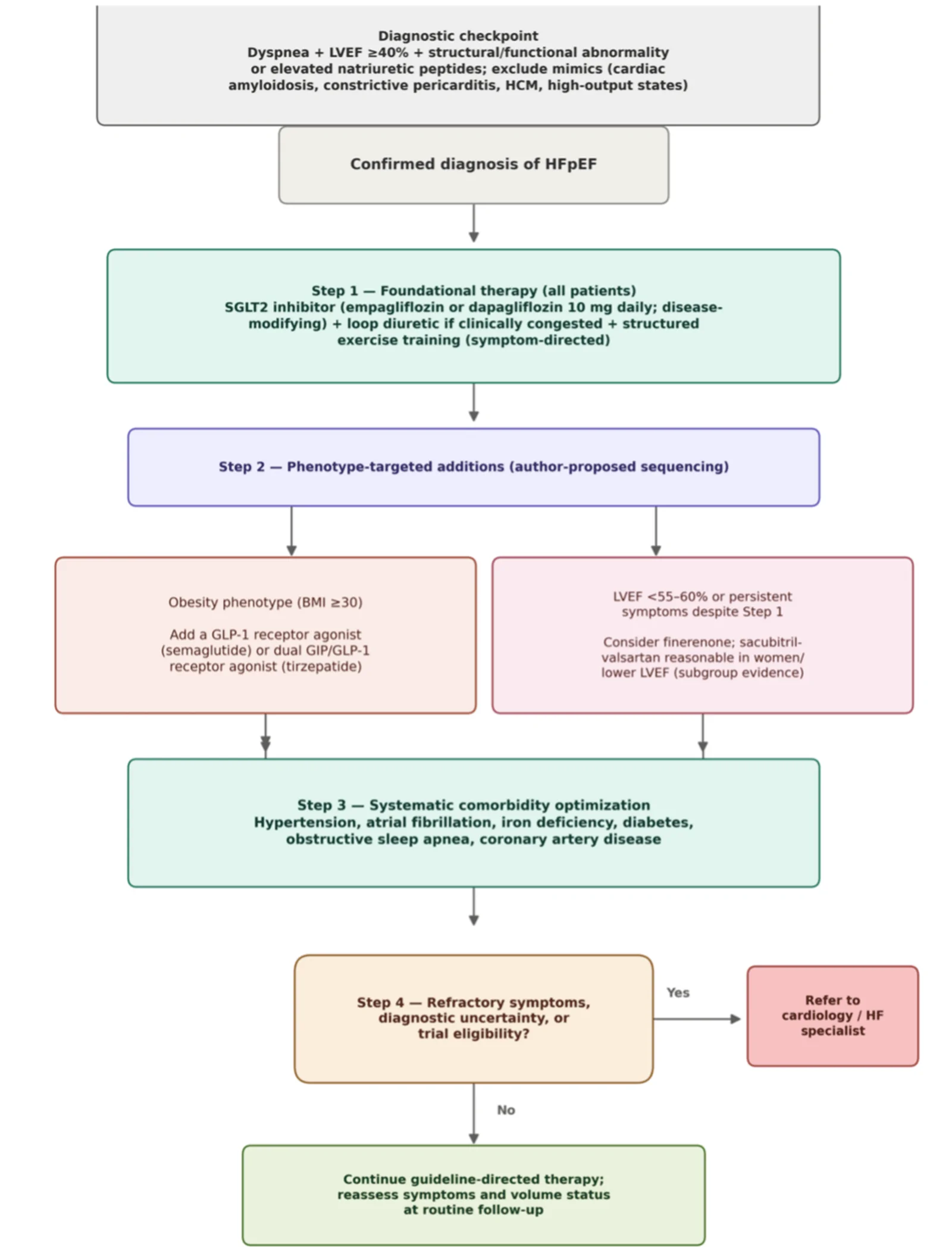
Diagnostic checkpoint and stepwise, phenotype-guided treatment framework for heart failure with preserved ejection fraction (HFpEF) GLP-1: glucagon-like peptide-1, GIP: glucose-dependent insulinotropic polypeptide, HCM: hypertrophic cardiomyopathy, LVEF: left ventricular ejection fraction, SGLT2: sodium-glucose cotransporter-2. Image Credit: This figure was created by the authors using Matplotlib, Version 3.10 (Python, Python Software Foundation, Wilmington, DE, USA). Note: This is an author-derived, evidence-informed framework, not a prospectively validated or guideline-endorsed treatment sequence.

The proposed treatment framework comprises four sequential steps: universal foundational therapy, phenotype-targeted additions, systematic comorbidity optimization, and defined referral triggers. This sequence (Figure 1) is an evidence-informed synthesis by the authors rather than a prospectively validated or guideline-endorsed treatment algorithm; where the sequence diverges from or extends beyond the 2026 ACC ECDP, this is noted explicitly.

Step 1: Universal foundational therapy (all patients with HFpEF)

SGLT2 inhibitors are disease-modifying, foundational therapy for HFpEF, with trial evidence supporting effects on hospitalization and cardiovascular death; loop diuretics and exercise training, by contrast, are symptom-directed and have not been shown to reduce hospitalization or mortality. The EMPEROR-Preserved trial (empagliflozin, n = 5,988) and the DELIVER trial (dapagliflozin, n = 6,263) each demonstrated an 18%-21% reduction in the composite of cardiovascular death or heart failure hospitalization, driven primarily by fewer hospitalizations, with consistent benefit regardless of diabetes status or baseline ejection fraction [7,8,11]. A comprehensive meta-analysis of five SGLT2 inhibitor trials (n = 21,947) confirmed reductions in cardiovascular death (hazard ratio (HR) 0.87), heart failure hospitalization (HR 0.72), and all-cause mortality (HR 0.92) across the ejection fraction spectrum [8].

In practice, empagliflozin 10 mg or dapagliflozin 10 mg daily should be prescribed for all patients with HFpEF lacking contraindications, as both agents carry FDA-approved indications for reducing the risk of cardiovascular death and heart failure hospitalization in adults with heart failure [12,13]. Contraindications and precautions include an estimated glomerular filtration rate (eGFR) below the threshold specified in each agent's prescribing information, a history of diabetic ketoacidosis, and volume depletion; these are distinct from the stability criteria used in the trials of in-hospital initiation discussed below, which were eligibility criteria for those specific studies rather than formal prescribing requirements. Initiation is supported by trial data during hospitalization for acute decompensated heart failure once the patient is hemodynamically stable; the criteria used in these trials, such as a systolic blood pressure of 100 mmHg or higher, no intravenous inotropes for 24 hours or longer, and an eGFR of 20 mL/min/1.73 m² or higher, reflect the enrollment criteria of the underlying studies rather than universal eligibility thresholds, and meta-analyses of in-hospital initiation have demonstrated reductions in all-cause death and worsening heart failure events under these study conditions [8]. Clinicians should monitor for genital mycotic infections and reassess diuretic requirements, which may decrease after initiation [11].

Loop diuretics are symptom-directed therapy for patients with clinical congestion; they have not been shown to reduce hospitalization or mortality in HFpEF, and should be titrated to achieve and maintain euvolemia rather than initiated routinely in euvolemic patients. The 2022 AHA/ACC/HFSA guideline supports diuretic use for symptom relief in congested patients [6]. The TRANSFORM-HF trial (n = 2,859) showed no meaningful difference between furosemide and torsemide in all-cause mortality, supporting individualized selection [14]. Thiazide diuretics may be added for resistant congestion with careful electrolyte monitoring. Higher loop diuretic doses are associated with worse outcomes in observational studies, though this likely reflects disease severity rather than a causal effect, and SGLT2 inhibitors augment natriuresis and may reduce diuretic requirements [11].

Supervised exercise training is a symptom-directed intervention that has consistently demonstrated clinically meaningful improvements in peak oxygen consumption, six-minute walk distance, and quality of life in patients with HFpEF, with effect sizes comparable to or exceeding those seen in HFrEF; it has not been shown to reduce hospitalization or mortality [4]. Both moderate-intensity continuous training and high-intensity interval training are reasonable options, and the 2022 AHA/ACC/HFSA guideline recommends at least 150 minutes per week of moderate-intensity aerobic activity [6]. Medicare cardiac rehabilitation coverage has historically been more clearly established for HFrEF than for HFpEF; clinicians should advocate for coverage where appropriate and, when formal programs are unavailable, prescribe structured home-based exercise tailored to functional status and frailty.

Step 2: Phenotype-targeted additions (author-proposed sequencing)

The heterogeneity of HFpEF demands a phenotype-guided approach to additional therapies. The additions below, and the order in which they are presented, reflect the authors' proposed sequencing rather than a trial-validated or guideline-mandated order; the 2026 ACC ECDP designates SGLT2 inhibitor plus nonsteroidal MRA as foundational (not sequential) therapy for most patients, with incretin-based therapy layered on for the obesity phenotype, an emphasis that differs somewhat from the step 2 framing used here [5]. With that caveat, additions should be considered based on clinical phenotype, beginning with obesity and extending to lower ejection fraction or persistent symptoms.

For patients with an obesity phenotype (body mass index of 30 kg/m² or higher), GLP-1 receptor agonists and dual GIP/GLP-1 receptor agonists represent a major therapeutic advance, and the 2026 ACC ECDP now recommends this approach directly [5]. The STEP-HFpEF trial (semaglutide 2.4 mg weekly, n = 529) demonstrated improvements in the Kansas City Cardiomyopathy Questionnaire Clinical Summary Score (KCCQ-CSS; 16.6 points with semaglutide versus 8.7 with placebo, estimated treatment difference (ETD) 7.8 points, 95% confidence interval (CI) 4.8-10.9), six-minute walk distance (21.5 m versus 1.2 m, ETD 20.3 m), body weight (-13.3% versus -2.6%, ETD -10.7 percentage points), and C-reactive protein (treatment ratio 0.61) [10]. These are functional and symptomatic outcomes; STEP-HFpEF was not powered for and did not demonstrate a reduction in hospitalization or mortality. The companion STEP-HFpEF DM trial (n = 616) showed smaller but consistent benefits in patients with type 2 diabetes. A pooled analysis of four trials (SELECT, FLOW, STEP-HFpEF, and STEP-HFpEF DM; n = 3,743 with HFpEF) showed a 31% reduction in cardiovascular death or worsening heart failure events (HR 0.69); notably, SELECT and FLOW were not HFpEF-specific trials, which should be considered when interpreting this pooled estimate [15]. The SUMMIT trial (tirzepatide up to 15 mg weekly, n = 731) demonstrated a 38% reduction in cardiovascular death or worsening heart failure (HR 0.62, 95% CI 0.41-0.95), with improvements in KCCQ-CSS (6.9 points), six-minute walk distance (18.3 m), and body weight (-11.6%) over a median follow-up of 104 weeks [2].

Neither agent is currently FDA-approved specifically for HFpEF. Semaglutide (Wegovy) is approved for chronic weight management and cardiovascular risk reduction in adults with established cardiovascular disease and obesity or overweight, and tirzepatide (Zepbound) is approved for chronic weight management and moderate-to-severe obstructive sleep apnea in adults with obesity; both should be prescribed for these approved indications in patients with HFpEF, with the additional heart failure benefit regarded as a secondary consideration [16,17]. This is distinct from the SUMMIT and STEP-HFpEF trial eligibility criteria, which are not themselves formal prescribing requirements. Important limitations of the randomized trial evidence should also temper enthusiasm: the SUMMIT trial had a fragility index of 2 for the primary composite outcome, a numerical imbalance in cardiovascular death favoring placebo (10 versus 5 events), and a 12.2% withdrawal-of-consent rate, so these data warrant cautious interpretation pending larger, dedicated HFpEF outcomes trials [2].

For patients with LVEF below 55%-60% or with persistent symptoms despite foundational therapy, several agents have trial support, though the specific sequencing and threshold used here reflect author judgment harmonized with the 2026 ACC ECDP rather than a trial-established indication [5]. The FINEARTS-HF trial (finerenone, n = 6,001) demonstrated a 16% reduction in the composite of total worsening heart failure events and cardiovascular death (rate ratio 0.84, 95% CI 0.74-0.95) over a median follow-up of 32 months, with consistent benefit across the LVEF spectrum (LVEF 50%: rate ratio 0.84; LVEF 50%-60%: rate ratio 0.80; LVEF ≥ 60%: rate ratio 0.94; p-interaction = 0.70) and significant improvement in the KCCQ total symptom score [1]. FINEARTS-HF enrolled patients based on LVEF and elevated natriuretic peptides or recent decompensation; it did not select patients specifically for persistent symptoms after a trial of SGLT2 inhibitor therapy, so “add for persistent symptoms” in Figure 1 reflects a practical sequencing proposal, not a trial-defined indication. Finerenone (Kerendia) received FDA approval on July 14, 2025, with a labeled indication to reduce the risk of cardiovascular death, hospitalization for heart failure, and urgent heart failure visits in adults with heart failure and LVEF of 40% or higher [9]. The 2026 ACC ECDP now favors finerenone over spironolactone as the MRA of choice, given its more favorable hyperkalemia profile, and positions the SGLT2 inhibitor plus MRA combination as foundational rather than sequential [5]. In practice, finerenone (maximum dose 20 mg or 40 mg daily) can be added to SGLT2 inhibitor therapy, with efficacy appearing consistent regardless of concomitant SGLT2 inhibitor use [1]. Potassium should be monitored closely, as hyperkalemia risk is increased, though this may be mitigated by concomitant SGLT2 inhibitor use [1].

PARAGON-HF (sacubitril-valsartan, n = 4,822) did not meet its primary endpoint of reduced total heart failure hospitalizations and cardiovascular death (rate ratio 0.87, 95% CI 0.75-1.01, p = 0.06); this was a neutral trial. Prespecified subgroup analyses suggested greater benefit in patients with LVEF of 57% or lower and in women, but these are hypothesis-generating subgroup findings and should not be presented as an established treatment indication [4,18]. Sacubitril-valsartan (Entresto) carries a broad FDA indication for reducing the risk of cardiovascular death and heart failure hospitalization in adult patients with chronic heart failure, and the 2022 AHA/ACC/HFSA guideline provides a Class IIb recommendation on the basis of this subgroup evidence [6]. The 2026 ACC ECDP similarly positions sacubitril-valsartan as reasonable in women and in patients with LVEF below 55%-60%, based on the same PARAGON-HF subgroup data, with an angiotensin receptor blocker as an alternative when sacubitril-valsartan is unavailable or contraindicated [5].

Spironolactone may be considered in selected patients, particularly those with LVEF at the lower end of the HFpEF spectrum, based on TOPCAT Americas data (n = 3,445); however, the 2026 ACC ECDP now favors finerenone over spironolactone when a nonsteroidal MRA is an option, given finerenone's more favorable hyperkalemia profile [5,19]. Mineralocorticoid receptor antagonists have more broadly been shown to reduce cardiovascular death or heart failure hospitalization across the ejection fraction spectrum [1,4]. Spironolactone should be used with caution regarding hyperkalemia, especially in the absence of a concomitant SGLT2 inhibitor.

Step 3: Systematic comorbidity optimization

Comorbidity management is integral to HFpEF care and should be addressed in parallel with pharmacotherapy. Blood pressure should be individualized rather than targeted to a single fixed number: the 2026 ACC ECDP identifies a systolic blood pressure of 120-129 mmHg as an optimal range for most patients, while the 2022 AHA/ACC/HFSA guideline supports a general target near 130/80 mmHg [5,6]. In older patients or those with frailty, orthostatic symptoms, or limited blood-pressure reserve, a less aggressive, individualized target should be used, with renin-angiotensin-aldosterone system inhibitors as reasonable first-line agents given trial experience. For atrial fibrillation, a rhythm-control strategy, whether catheter ablation or antiarrhythmic therapy, should be considered early; aggressive rate control should be avoided, as it may worsen exercise intolerance given the dependence of stroke volume on heart rate in this population [4]. Iron deficiency, present in up to 50% of patients with heart failure, contributes to exercise intolerance independent of anemia and should be screened for with ferritin and transferrin saturation [4]. HFpEF-specific evidence for treating iron deficiency is limited and does not carry the weight of the larger HFrEF iron trials [4]; intravenous iron may be considered in symptomatic, iron-deficient patients with HFpEF, but this recommendation rests on a substantially smaller evidence base than the corresponding HFrEF recommendation. In patients with diabetes, SGLT2 inhibitors should be used as first-line glucose-lowering therapy, with GLP-1 receptor agonists providing additional cardiometabolic benefit consistent with the comorbidity-focused approach of the 2026 ACC ECDP [5]. Obstructive sleep apnea should be screened for and treated, particularly in patients with obesity, and coronary artery disease, common in patients with HFpEF, should be evaluated for, since complete revascularization is associated with improved outcomes in observational studies.

Step 4: Referral triggers

Referral to cardiology or a heart failure specialist should be considered in several circumstances: diagnostic uncertainty despite the checkpoint above, including persistent consideration of HFpEF mimics such as cardiac amyloidosis, constrictive pericarditis, hypertrophic cardiomyopathy, or high-output states; refractory symptoms despite optimized step 1-3 therapy; consideration of advanced diagnostics such as invasive hemodynamics, cardiac magnetic resonance imaging, or endomyocardial biopsy; potential clinical trial eligibility, for example, the HERMES trial of ziltivekimab targeting interleukin-6 (NCT05636176) or the SPIRIT-HF (NCT04727073) and SPIRRIT (NCT02901184) trials of spironolactone [4]; and evaluation for device-based therapies, including pulmonary artery pressure monitoring or investigational atrial shunt devices, none of which have shown consistent benefit to date [5].

Table 1 summarizes evidence for individual therapies; it does not imply that the sequence or combination shown in Figure 1 has itself been validated.

**Table 1:** Summary evidence table for HFpEF pharmacotherapy and exercise training CI: confidence interval, CV: cardiovascular, eGFR: estimated glomerular filtration rate, HF: heart failure, HFpEF: heart failure with preserved ejection fraction, hosp: hospitalization, HR: hazard ratio, K⁺: potassium, KCCQ-CSS: Kansas City Cardiomyopathy Questionnaire Clinical Summary Score, LVEF: left ventricular ejection fraction, OSA: obstructive sleep apnea, RCT: randomized controlled trial, RR: rate ratio, VO₂: oxygen consumption, 6MWD: six-minute walk distance.

Therapy	Key trial(s)	n	Primary result	FDA/HF-specific approval status	Practical pearl	Ref
Empagliflozin 10 mg daily	EMPEROR-Preserved	5,988	21% ↓ CV death/HF hosp (HR 0.79)	HF-specific: approved	Diuretic only if congested; reassess dose	[7]
Dapagliflozin 10 mg daily	DELIVER	6,263	18% ↓ CV death/HF hosp (HR 0.82)	HF-specific: approved	eGFR ≥ 20 was a trial criterion, not a label cutoff	[11]
Finerenone 20-40 mg daily	FINEARTS-HF	6,001	16% ↓ worsening HF/CV death (RR 0.84)	HF-specific: approved, LVEF ≥ 40% (Jul 2025)	Monitor K⁺; preferred over spironolactone per 2026 ACC ECDP	[1]
Sacubitril-valsartan	PARAGON-HF	4,822	Neutral primary endpoint (RR 0.87, 95% CI 0.75-1.01, p = 0.06); subgroup signal in LVEF ≤ 57%, women	Approved for chronic HF generally, not HFpEF-specific	Subgroup evidence only; not an established HFpEF indication	[18]
Spironolactone	TOPCAT	3,445	Primary endpoint not met; ↓ HF hosp in Americas cohort only	Approved; not HFpEF-specific	2026 ACC ECDP now favors finerenone	[19]
Semaglutide 2.4 mg weekly	STEP-HFpEF	529	KCCQ-CSS ETD 7.8 pts; weight ETD -10.7 pts; 6MWD ETD 20.3 m (functional/symptomatic outcomes)	Not HF-specific; approved for obesity, CV risk reduction	HF benefit is a secondary consideration, not the approved indication	[10]
Tirzepatide up to 15 mg weekly	SUMMIT	731	HR 0.62 for CV death/worsening HF; KCCQ-CSS +6.9 pts	Not HF-specific; approved for obesity, OSA	Fragility index of 2; await larger outcomes trials	[2]
Loop diuretics	TRANSFORM-HF	2,859	No difference furosemide vs. torsemide (all-cause mortality)	Approved; symptom indication only	Use if congested; not a mortality/hospitalization therapy	[14]
Exercise training	Multiple RCTs, meta-analyses	Varies by study	Peak VO₂ +1.85 mL/min/kg; 6MWD +33 m (functional outcomes)	N/A	Medicare does not cover cardiac rehab for HFpEF	[4]

## Discussion

This framework is intended as a concise companion to, not a replacement for, the 2026 ACC ECDP, which is the current authoritative guideline-level source for HFpEF management [5]. The two align on several major points: SGLT2 inhibitors and nonsteroidal MRAs as cornerstone therapy, incretin-based therapy for the obesity phenotype, and caution around routine beta-blocker use. They differ in framing in a few respects that clinicians should be aware of. First, the 2026 ACC ECDP treats the SGLT2 inhibitor and MRA combination as foundational and largely concurrent, whereas this report's step 1/step 2 structure presents MRA therapy as an addition; in practice, clinicians following the ACC ECDP may reasonably initiate both classes together in appropriate patients rather than sequentially. Second, the ACC ECDP favors finerenone over spironolactone as a default, a preference incorporated into this report's discussion of spironolactone above. Third, the ACC ECDP's blood pressure target (120-129 mmHg) differs modestly from the general target cited from the 2022 AHA/ACC/HFSA guideline (near 130/80 mmHg); both should be individualized in older or frail patients. Finally, the diagnostic pathway summarized in Figure 1 and described above draws directly on the ACC ECDP's H2FPEF/HFA-PEFF sequential scoring approach and its list of mimics to exclude, rather than proposing an independent diagnostic method.

Several pitfalls recur in HFpEF management and warrant emphasis alongside the framework above. Beta-blockers are frequently used routinely despite lacking evidence to support this practice in HFpEF; an individual patient-level meta-analysis of 11 randomized heart failure trials did not demonstrate benefit for patients with HFpEF. Their use should be reserved for comorbid indications such as prior myocardial infarction, angina, or atrial fibrillation requiring rate control, consistent with the 2026 ACC ECDP [4,5,20]. Delaying SGLT2 inhibitor initiation until outpatient follow-up risks therapeutic inertia, since trial evidence supports starting these agents during hospitalization once the patient meets the stability criteria used in those trials, with meta-analyses showing a mortality reduction associated with in-hospital initiation under those conditions [8]. Strict sodium restriction, such as a target of 1,500 mg per day, lacks consistent supporting evidence in HFpEF, and overly restrictive diets may reduce palatability and nutritional intake, whereas moderate sodium reduction remains reasonable. HFpEF mimics, including cardiac amyloidosis, constrictive pericarditis, hypertrophic cardiomyopathy, and high-output states, can present identically to HFpEF and require specific therapies, so the diagnostic checkpoint described above and a high index of suspicion are essential. Finally, the obesity phenotype, present in a large proportion of patients with HFpEF, is often neglected despite the availability of GLP-1 receptor agonists and dual GIP/GLP-1 receptor agonists, which offer meaningful symptomatic and functional improvement beyond weight loss alone in eligible patients [2].

Several ongoing trials will further refine HFpEF management. The HERMES trial is evaluating ziltivekimab, an anti-interleukin-6 agent, in HFpEF with systemic inflammation (NCT05636176), and the SPIRIT-HF (NCT04727073) and SPIRRIT (NCT02901184) trials are evaluating spironolactone in HFpEF [4]. Additional finerenone outcomes trials across the heart failure phenotype spectrum, and dedicated outcomes trials of semaglutide and tirzepatide for HFpEF-specific indications, are anticipated as the regulatory and evidentiary landscape continues to evolve [4].

Lifetime benefit modeling projects that comprehensive combination therapy, comprising an SGLT2 inhibitor plus finerenone with or without sacubitril-valsartan in selected patients, may extend event-free survival by 3.6 to 4.9 years in a 65-year-old patient, with meaningful projected gains across ages 55 to 85 [3]. These are modeled estimates, not observed clinical outcomes: they are derived from cross-trial analyses of DELIVER, FINEARTS-HF, and PARAGON-HF rather than observed outcomes from a single trial of combination therapy, and they assume independent, additive treatment effects that have not been prospectively confirmed. They reinforce the general rationale for early, comprehensive treatment initiation, but should not be read as a demonstrated benefit of the specific sequence proposed in this report.

Several limitations of this technical report should be acknowledged. First, and most importantly, the stepwise treatment framework presented here is an evidence-informed synthesis by the authors rather than a guideline-endorsed or prospectively validated treatment sequence; no prospective trial has validated a specific order of therapy initiation in HFpEF, and the demonstrated clinical benefits described throughout this report belong to the individual therapies studied in their respective trials, not to the sequence as a whole. Second, the lifetime benefit estimates for combination therapy are modeled, cross-trial statistical projections rather than observed outcomes [3]. Third, the phenotype-guided approach, such as the obesity phenotype and lower LVEF phenotype, is supported by subgroup analyses and biological plausibility but has not been prospectively validated in a trial randomizing patients to phenotype-matched versus unmatched therapy. Fourth, the evidence base for incretin-based therapies in HFpEF, while promising, is limited by relatively small sample sizes, a short follow-up for some endpoints, and low fragility indices for event-driven outcomes [2]. Fifth, this report focuses on pharmacologic and exercise-based interventions and does not comprehensively address device-based therapies, which remain investigational. Finally, the rapidly evolving evidence landscape means that some recommendations, including this report's, may require updating as results from ongoing trials and future guideline revisions become available.

## Conclusions

HFpEF is no longer a condition without effective therapies. The individual components of the framework presented here, including SGLT2 inhibitor therapy, diuretics for congestion, incretin-based therapy for obesity, finerenone, sacubitril-valsartan in appropriate subgroups, and structured comorbidity management, are each supported by randomized trials or, in some cases, subgroup or observational evidence, and can meaningfully reduce hospitalizations, improve symptoms, or both, depending on the therapy. The stepwise sequence in which these components are presented is the authors' proposed synthesis, situated alongside the 2026 ACC ECDP, and has not itself been prospectively validated; it should be understood as a practical organizing framework rather than a demonstrated treatment strategy in its own right.

## References

[1] Solomon SD, McMurray JJ, Vaduganathan M (2024). Finerenone in heart failure with mildly reduced or preserved ejection fraction. N Engl J Med.

[2] Packer M, Zile MR, Kramer CM (2025). Tirzepatide for heart failure with preserved ejection fraction and obesity. N Engl J Med.

[3] Vaduganathan M, Claggett BL, Chatur S (2026). Lifetime benefits of comprehensive medical therapy in heart failure with mildly reduced or preserved ejection fraction. Nat Med.

[4] Campbell P, Rutten FH, Lee MM, Hawkins NM, Petrie MC (2024). Heart failure with preserved ejection fraction: everything the clinician needs to know. Lancet.

[5] Kittleson MM, Panjrath GS, Bates K, Breathett KK, Dixon DL, Januzzi JL Jr, Mohammed SF (2026). Management of heart failure with preserved ejection fraction: 2026 ACC expert consensus decision pathway: a report of the American College of Cardiology Solution Set Oversight Committee. J Am Coll Cardiol.

[6] Heidenreich PA, Bozkurt B, Aguilar D (2022). 2022 AHA/ACC/HFSA guideline for the management of heart failure: a report of the American College of Cardiology/American Heart Association Joint Committee on Clinical Practice Guidelines. J Am Coll Cardiol.

[7] Anker SD, Butler J, Filippatos G (2021). Empagliflozin in heart failure with a preserved ejection fraction. N Engl J Med.

[8] Vaduganathan M, Docherty KF, Claggett BL (2022). SGLT-2 inhibitors in patients with heart failure: a comprehensive meta-analysis of five randomised controlled trials. Lancet.

[9] (2025). Food and Drug Administration (FDA). KERENDIA (finerenone) tablets, for oral use (prescribing information). Bayer HealthCare Pharmaceuticals, Whippany, NJ. Revised July.

[10] Kosiborod MN, Abildstrøm SZ, Borlaug BA (2023). Semaglutide in patients with heart failure with preserved ejection fraction and obesity. N Engl J Med.

[11] Solomon SD, McMurray JJ, Claggett B (2022). Dapagliflozin in heart failure with mildly reduced or preserved ejection fraction. N Engl J Med.

[12] (2025). Food and Drug Administration (FDA). JARDIANCE® (empagliflozin tablets), for oral use (prescribing information). Revised.

[13] (2023). Food and Drug Administration (FDA). FARXIGA® (dapagliflozin) tablets, for oral use (prescribing information). Revised.

[14] Mentz RJ, Anstrom KJ, Eisenstein EL (2023). Effect of torsemide vs furosemide after discharge on all-cause mortality in patients hospitalized with heart failure: the TRANSFORM-HF randomized clinical trial. JAMA.

[15] Kosiborod MN, Deanfield J, Pratley R (2024). Semaglutide versus placebo in patients with heart failure and mildly reduced or preserved ejection fraction: a pooled analysis of the SELECT, FLOW, STEP-HFpEF, and STEP-HFpEF DM randomised trials. Lancet.

[16] (2025). Food and Drug Administration (FDA). WEGOVY (semaglutide) injection, for subcutaneous use (prescribing information). Novo Nordisk, Plainsboro, NJ. Revised.

[17] (2024). Food and Drug Administration (FDA). ZEPBOUND® (tirzepatide) Injection, for subcutaneous use (prescribing information). Revised.

[18] Solomon SD, McMurray JJ, Anand IS (2019). Angiotensin-neprilysin inhibition in heart failure with preserved ejection fraction. N Engl J Med.

[19] Pitt B, Pfeffer MA, Assmann SF (2014). Spironolactone for heart failure with preserved ejection fraction. N Engl J Med.

[20] Cleland JG, Bunting KV, Flather MD (2018). Beta-blockers for heart failure with reduced, mid-range, and preserved ejection fraction: an individual patient-level analysis of double-blind randomized trials. Eur Heart J.

